# Visual-Attentional Load Unveils Slowed Processing Speed in Multiple Sclerosis Patients: A Pilot Study with a Tablet-Based Videogame

**DOI:** 10.3390/brainsci10110871

**Published:** 2020-11-18

**Authors:** Marco Pitteri, Caterina Dapor, Stefano Ziccardi, Maddalena Guandalini, Riccardo Meggiato, Massimiliano Calabrese

**Affiliations:** Neurology Section, Department of Neuroscience, Biomedicine and Movement Sciences, University of Verona, 37134 Verona, Italy; caterina.dapor@univr.it (C.D.); stefano.ziccardi@univr.it (S.Z.); maddalena.guandalini@univr.it (M.G.); rmeggiato@gmail.com (R.M.)

**Keywords:** multiple sclerosis, speed of processing, visual-attentional load, videogame, computerized assessment

## Abstract

Slowing in information processing speed (IPS) is the key cognitive deficit in multiple sclerosis (MS). Testing IPS in different cognitive load conditions by using computerized tools might reveal initial IPS slowness underestimated by classic paper-and-pencil tests. To investigate the extent to which IPS can be affected by increased task demands, we developed three tasks based on the manipulation of the visual-attentional load, delivered with a home-made, tablet-based videogame. Fifty-one patients with MS (pwMS), classified as having no cognitive impairment in classic paper-and-pencil tests, and 20 healthy controls (HC) underwent the videogame tasks; reaction times (RTs) and accuracy were recorded. A significant reduced performance of pwMS as compared with HC was found on the videogame tasks, with pwMS being on average slower and less accurate than HC. Furthermore, pwMS showed a significantly more pronounced decrement in accuracy as a function of the visual-attentional load, suggesting a higher susceptibility to increased task demands. Significant correlations among the Symbol Digit Modalities Test (SDMT) and the videogame mean RTs and accuracy were found, providing evidence for the concurrent validity of the videogame as a valid tool to test IPS in pwMS. The high potential that might derive from the adoption of computerized assessment tools in clinical practice should be taken into consideration and investigated further.

## 1. Introduction

Multiple sclerosis (MS) is an inflammatory neurodegenerative disease of the central nervous system, characterized by white matter (WM) and grey matter (GM) damage [1]. Cognitive impairment (CI) is among the main sequalae of MS and its importance over the course of the disease has been highlighted in the past years. Up to half of the people with MS (pwMS) suffer from cognitive deficits as a consequence of both WM and GM damage [2]. CI has been reported in all stages and subtypes of MS and has been found to exert negative effects on employment, activities of daily living, and quality of life [3,4]. CI might help in identifying patients at higher risk of disease progression in the long term [5,6], an aspect of paramount importance from a clinical perspective [7]. Crucially, the detection of initial CI in pwMS might be difficult, especially by using only paper-and-pencil tests [7,8]. It has been reported that various compensation phenomena (e.g., neural plasticity, cognitive reserve) can contribute in masking initial changes in cognitive efficiency, thus limiting the possibility to early detect CI in pwMS [9,10,11].

By contrast, computer-based assessment has been reported to offer a greater sensitivity than paper-and-pencil test in the detection of subtle signs of cognitive disfunctions in different clinical populations (e.g., stroke, dementia, MS) [12,13,14,15,16]. With reference to MS, a growing literature is highlighting the advantage that might derive from the adoption of computerized testing, especially in the screening of information processing speed (IPS) and attention [17,18,19,20,21]. Accordingly, in a recent study by our research group [14] we developed a home-made, tablet-based videogame task of IPS. The task was administered to a group of pwMS classified as cognitive-preserved on the paper-and-pencil tests and to a group of healthy controls (HC). By recording participants’ reaction times (RTs), the videogame task successfully highlighted reduced IPS in pwMS as compared with HC. Interestingly, pwMS were found slower than HC on the videogame task, even though their performance on the Symbol Digit Modalities Test (SDMT) was classified as apparently preserved (i.e., z-scores > −1.5) [14].

Reduced IPS is the earliest and most common cognitive deficit in MS [22], and it has been reported that it might be linked to increasing task demand conditions [21,23]. Therefore, assessing IPS in increasing cognitive load conditions might help in identifying signs of IPS inefficiencies that, otherwise, would be neglected. Introducing additional cognitive requests (e.g., increasing the perceptual characteristics of the stimuli, adding concurrent tasks to be performed) reduces the cognitive resources available to effectively perform the task at hand, resulting in a decrement in the speed of execution and/or accuracy. Furthermore, it has been suggested that an increased cognitive load could limit the compensatory strategies of pwMS, with a detrimental effect on their cognitive efficiency [24]. In fact, when the cognitive demand is too high, a “saturation effect” occurs on the compensatory mechanisms, thus leading to clinically evident CI [24]. Computerized tasks developed according to cognitive load manipulation might detect early slowing in IPS, which might reveal subclinical cognitive inefficiency related to an underlying progression of the disease.

Grounding on the results of our previous study [14], with the present study we aimed to further investigate the extent to which IPS can be affected by increased cognitive load conditions. Therefore, we developed different videogame levels based on the manipulation of visual-attentional load. We adopted a mixed design to assess whether pwMS might be more susceptible to an increasing load than a group of neurologically HC. Our hypothesis was to observe a reduced IPS (in terms of slowed RTs and/or reduced accuracy) as a function of load in both pwMS and HC. However, we expected to observe a more pronounced effect in pwMS as compared with HC. Our secondary aim was to investigate the concurrent validity of the videogame levels with reference to the gold standard of IPS assessment in pwMS, that is the SDMT.

## 2. Materials and Methods

### 2.1. Study Sample

Fifty-one pwMS (mean age: 36.7 ± 9.1 years; mean education: 14.0 ± 3.0 years; F = 38; mean disease duration: 8.3 ± 6.6 years) were recruited at the MS Center of the Verona University Hospital (Verona, Italy). Inclusion criteria were diagnosis of relapsing remitting MS [25], no concomitant neurological (other than MS) or psychiatric conditions, no substance abuse, normal or corrected-to-normal vision, no relapses in the previous 6 months from enrollment, and no evidence of CI. The CI classification was based on a battery of neuropsychological tests carried out by experienced neuropsychologists, composed of the Brief Repeatable Battery of Neuropsychological Tests [26] and the Stroop Test [27]. In order to be classified as cognitive preserved, pwMS had to fail less than three neuropsychological tests [28].

At the time of the videogame testing, 11 pwMS were untreated, whereas 24 were treated with dimethyl-fumarate, 6 with fingolimod, 2 with natalizumab, 2 with teriflunomide, 1 with interferon beta-1a, 2 with cladribine, and 3 with ocrelizumab. Physical disability was measured with the Expanded Disability Status Scale (EDSS; [29] median = 1.5; range: 0–3.5).

A group of 20 neurologically HC (mean age: 34.3 ± 12.3 years; mean education: 17.5 ± 2.5 years; F = 7) took part in the present study as control group. Inclusion criteria for HC consisted of absence of neurological or psychiatric conditions, no substance abuse or medications, and normal or corrected-to-normal vision. The HC group was selected to be age-matched with the pwMS group, as age has been found to be the demographic characteristic most crucial in influencing simple RTs [30].

The study was approved by the local Ethic Committee, and all participants provided written informed consent prior to participation in the study.

### 2.2. The Videogame

#### 2.2.1. Graphics

The videogame is based on two-dimensional (2D) graphics. All the software graphics were created using the Gimp software (www.gimp.org, version 2.9.2), a cross-platform, open-source image editor. Stimuli were designed by an expert designer and were composed of three frames each, which has been proven to be the optimal compromise between fluidity and reactivity with respect to user interaction.

#### 2.2.2. Programming

The software was written in C and Java languages, then the Gamemaker (https://www.yoyogames.com/gamemaker, version 2) videogame engine was used. Gamemaker is an integrated development environment specialized in the display of high-speed 2D graphics and includes its own scripting language, GML. Afterward, the algorithms were easily converted in GML, while the graphics core comes from the engine’s kernel itself. The Gamemaker engine is for multiplatform development, so although the software runs actually in Android operating system, it could be easily converted for other operating systems as well (i.e., “iOS” or “Windows”).

#### 2.2.3. Hardware

The videogame ran on touch screen tablets Nvidia Shield with the following technical specifications: dimensions = 221 × 126 × 9.2 mm; weight = 390 g; display type = capacitive touchscreen, 16M colors; display size =185.6 cm^2^ (~66.6% screen-to-body ratio); display resolution = 1920 × 1200 pixels, 16:10 ratio (~283 ppi density); memory storage = 16 GB; RAM = 2 GB; processor = Nvidia Tegra K1; operating system = Android.

### 2.3. Stimuli Description

Three levels were developed according to an increasing visual-attentional load principle: (1) low, (2) medium, and (3) high visual-attentional load.

**Figure 1:**
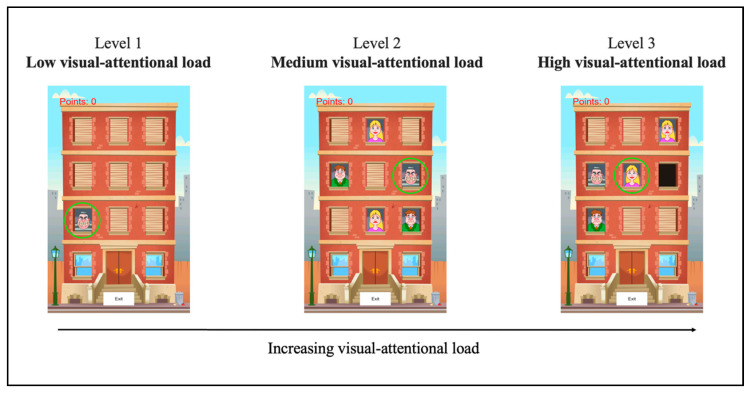
Example of the three videogame levels. (Note: the green circles have been added for illustrative purposes only to indicate the target that the participants were asked to touch in each level.)

In all three levels, the scenario consisted of an apartment building with nine windows (see Figure 1). Behind each window, a figure appeared which could be a prisoner, a woman, a man, or an empty window; the stimuli representing the woman and the man might have a happy face or a sad one (see Figure 1). According to the instruction of each level, participants were asked to touch one of the figures, as quickly as possible, as it appeared on the screen. The RTs, measured in milliseconds (ms), and the number of correct responses on each trial, and for each level, were recorded.

#### 2.3.1. Level 1: Low Visual-Attentional Load (Named “Low”)

Level 1 (low) was conceived to be a measure of how fast a subject is able to respond in the easiest condition, that is, low-attention-demanding. In this level, each trial started with only one window opening; behind the window, the target (the prisoner) appeared straight away. The window remained open for a maximum duration of 1000 ms. Participants were asked to touch the target as quickly as possible. The position of the target (i.e., the window in which it appeared) was randomized across trials.

#### 2.3.2. Level 2: Medium Visual-Attentional Load (Named “Medium”)

Level 2 (medium) was developed according to the same characteristics of level 1 (low), with the only difference being that the target appeared together with distractors. In this level, each trial started with three or five windows opening simultaneously. Behind one window, the target (the prisoner) appeared, while behind the other windows, two or four distractors were displayed, randomly chosen among women (happy and/or sad), men (happy and/or sad), and empty windows. The type (happy/sad woman, happy/sad man, or empty window) and the number (two or four) of distractors were randomized across trials; the position of both the target and the distractors were also randomly assigned. Like in level 1 (low), each window remained open for a maximum of 1000 ms; participants were asked to touch the target (the prisoner) as fast as possible. Once the participants touched the target (i.e., correct response), touched a distractor (i.e., error), or after the 1000 ms time limit passed (i.e., omission), all the windows closed simultaneously.

#### 2.3.3. Level 3: High Visual-Attentional Load (Named “High”)

In level 3 (high), in addition to the presence of distractors, the target was conceived to be more perceptively difficult to detect than the previous one (the prisoner). In this level, the target consisted of women or men that were *smiling*. Each trial started with three or five windows opening simultaneously, one showing the target (the happy man or the happy woman), and the others displaying the distractors. The type (woman or man) and the position of the target were randomized across trials; similarly, the type (sad woman, sad man, prisoner, or empty window), the number (two or four), and the position of the distractors were randomly assigned. As in the previous levels, each window remained open for a maximum of 1000 ms; participants were instructed to touch the target (i.e., happy woman or happy man) as quickly as possible. Once the participants touched the target (i.e., correct response), touched a distractor (i.e., error), or after the 1000 ms time limit passed (i.e., omission), all the windows closed simultaneously.

See Figure 1 for an illustrative example of the three videogame levels.

Each level of the videogame (i.e., low, medium, high) was composed of eighty trials. In the upper left corner of the screen, a score was displayed as a function of the points attributed to the responses given.

### 2.4. Procedure

Each participant was tested individually in a quiet room.

The videogame started with a 10-trial practice session (warm up) so as to familiarize the participants with the videogame setting, the scenario, the touch screen, the different characters, and the target of the first and the second levels (i.e., the prisoner). After the warm up, participants were administered the three levels in a fixed order: level 1 (low visual-attentional load), level 2 (medium visual-attentional load), and level 3 (high visual-attentional load). Between the second (medium) and the third (high) levels, participants familiarized themselves with the new target (i.e., happy man/woman) by performing a 10-trial practice session where only one window opened displaying a happy man or a happy woman. This was done in order to get participants washed out from the previous target (i.e., the prisoner) and to get used to the new one (i.e., man or woman smiling). After completing the third level (high), participants were asked to perform the first level (low) again, hereinafter named “low_bis_”, in order to assess whether a short-term depletion of cognitive resources over time might have influenced participants’ performance.

Overall, each videogame level lasted about one minute and a half; the whole videogame testing lasted no more than ten minutes.

Right before the videogame testing, pwMS were administered the oral version of the SDMT, in order to evaluate the presence of IPS impairment. The SDMT was chosen for this purpose as it is a widely recognized test of IPS that might serve as the gold standard for the assessment of IPS in pwMS [31]. The SDMT score was considered impaired if it fell below the 5th percentile, that is, whether the z score was less than −1.64 standard deviation (SD) with reference to the normative data [26].

### 2.5. Study Design

We adopted a mixed factorial design, which involved two independent variables. The between-participants independent variable was *Group* (two levels: pwMS vs. HC). The within-participant independent variable was *Load* (three levels: low, medium, and high).

The dependent variables were RTs and accuracy. 

### 2.6. Statistical Analyses

Mean RTs were subjected to a mixed repeated-measures ANOVA, with *Load* (low, medium, high) as the within-subjects factor and *Group* (HC vs. pwMS) as the between-subjects factor. Similarly, a mixed repeated-measures ANOVA was performed to test if the two groups differed on the number of correct responses given, with *Load* (low, medium, high) as the within-subjects factor and *Group* (HC vs. pwMS) as the between-subjects factor. Post hoc analyses with Holm–Bonferroni correction were used to further investigate the significant effects observed in these two ANOVAs performed on mean RTs and on accuracy through the three levels (low, medium, and high).

The assumption of sphericity was tested with the Mauchly test; it was met for mean RTs but not for accuracy. Therefore, the ANOVA on accuracy was performed with the Greenhouse–Geisser correction.

In order to exclude the effect of a short-term depletion of cognitive resources over time on the observed results, mean RTs and the total number of correct responses were subjected to mixed repeated-measures ANOVAs, with *Time* (low vs. low_bis_) as the within-subjects factor and *Group* (HC vs. pwMS) as the between-subjects factor.

Spearman’s correlation analyses between mean RTs and accuracy of each videogame level and the total number of correct responses on the SDMT were performed in order to obtain a measure of concurrent validity of each videogame task.

All numerical values are reported as mean ± SD. All the statistical analyses were performed with JASP (Version 0.13.1; JASP Team, 2020) [32].

## 3. Results

### 3.1. RTs

The mixed repeated-measures ANOVA on mean RTs revealed a main effect of *Load* (*F*(2,138) = 1134.507, *p* < 0.001, η^2^_p_ = 0.94). Post hoc analyses with Holm–Bonferroni correction revealed that mean RTs significantly increased as a function of increasing visual-attentional load in both pwMS (low: 508.5 ± 72.2 ms; medium: 631.1 ± 66.7 ms; high: 780.2 ± 37.3 ms) and HC (low: 462.3 ± 40.3 ms; medium: 581.0 ± 55.3 ms; high: 752.9 ± 29.0 ms).

A main effect of *Group* was also observed (*F*(1,69) = 9.732, *p* = 0.003, η^2^_p_ = 0.12). PwMS were found to be slower than HC on each level; the difference between the two groups was significant in the first (low; pwMS: 508.5 ± 72.2 ms; HC: 462.3 ± 40.3 ms) and in the second (medium; pwMS: 631.1 ± 66.7 ms; HC: 581.0 ± 55.3 ms) levels, but not in the third one (high; pwMS: 780.2 ± 37.3 ms; HC: 752.9 ± 29.0 ms).

**Figure 2:**
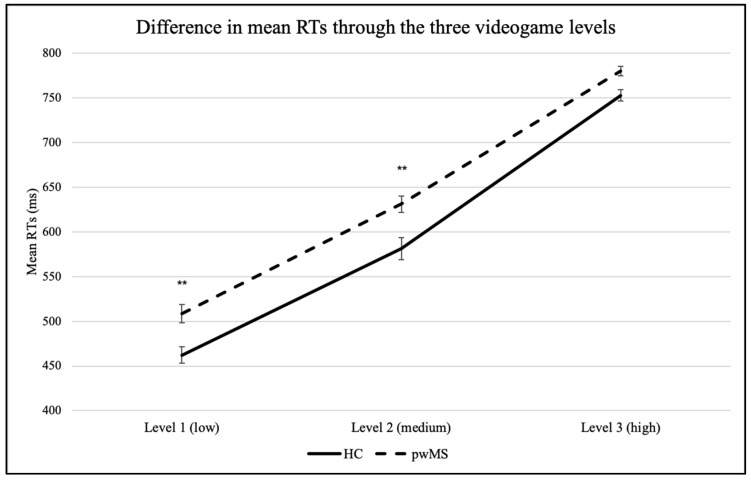
Mean reaction times (RTs) in the three videogame levels in healthy controls (HC) and patients with MS (pwMS). Notes: error bars indicate the standard error of the mean (SEM); ** *p* < 0.01.

No significant interaction Load*Group was found (*F*(2,138) = 2.130, *p* = 0.12, η^2^_p_ = 0.03): the increasing mean RTs as a function of visual-attentional load were similar in the two groups. See Figure 2.

### 3.2. Accuracy

The mixed repeated-measures ANOVA on correct responses showed a main effect of *Load* (*F*(1.176, 81.178) = 391.529, *p* < 0.001, η^2^_p_ = 0.85). Post hoc analyses with Holm–Bonferroni correction revealed that the number of correct responses significantly decreased in the third (high) level in comparison to the first (low) and to the second (medium) levels, in both pwMS (low: 77.5 ± 3.0; medium: 75.8 ± 5.8; high: 45.9 ± 13.3) and HC (low: 78.7 ± 1.5; medium: 77.9 ± 2.1; high: 55.4 ± 7.0). By contrast, no significant difference was observed between level 1 and level 2 in either groups (*p_s_* > 0.05).

A main effect of *Group* was also observed (*F*(1,69) = 7.541, *p* = 0.008, η^2^_p_ = 0.10). Post hoc analyses evidenced that the two groups did not significantly differ in the correct responses given in level 1 (low; pwMS: 77.5 ± 3.0; HC: 78.7 ± 1.5) and in level 2 (medium; pwMS: 75.8 ± 5.8; HC: 77.9 ± 2.1), but they significantly differed in the number of correct responses given in level 3 (high; pwMS: 45.9 ± 13.3; HC: 55.4 ± 7.0).

**Figure 3:**
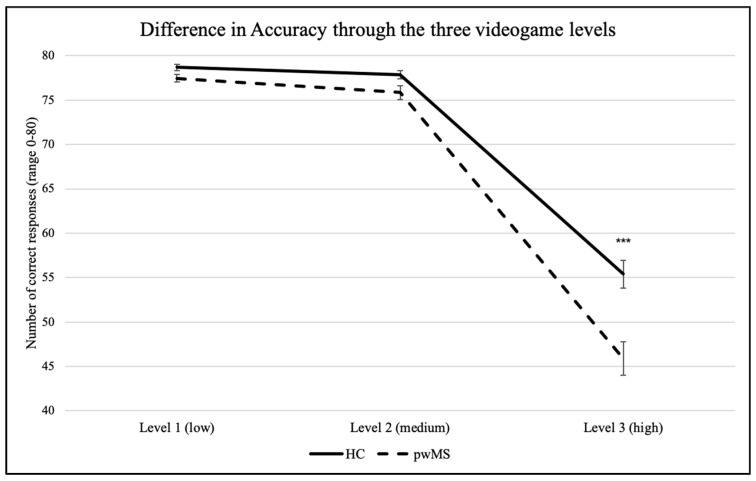
Number of correct responses in the three videogame levels in HC and pwMS. Note: error bars indicate the standard error of the mean (SEM); *** *p* < 0.001.

The interaction Load*Group was also significant (*F*(1.176,81.178) = 8.480, *p* = 0.003, η^2^_p_ = 0.11): the decrement in accuracy through the levels was significantly more pronounced in pwMS than in HC. See Figure 3.

### 3.3. Control Task for Short-Term Depletion of Cognitive Resources over Time

The mixed repeated-measures ANOVA on mean RTs revealed a main effect of *Time* (*F*(1,69) = 68.091, *p* < 0.001, η^2^_p_ = 0.05): mean RTs significantly decreased as a function of time in both pwMS (low: 508.5 ± 72.2; low_bis_: 470.9 ± 60.08) and HC (low: 462.3 ± 40.3; low_bis_: 435.5 ± 44.0). A main effect of *Group* was also observed, *F*(1,69) = 6.851, *p* = 0.011, η^2^_p_ = 0.09. On average, pwMS were slower than HC both at the beginning (low; pwMS: 508.5 ± 72.2; HC: 462.3 ± 40.3) and at the end (low_bis_; pwMS: 470.9 ± 60.08; HC: 435.4 ± 44.0) of the videogame administration. However, no significant interaction Time*Group was found, *F*(1,69) = 1.898, *p* = 0.17, η^2^_p_ = 0.001: the increment in performance (i.e., lower RTs) over time was similar in the two groups.

The mixed repeated-measures ANOVA on accuracy showed a main effect of *Time F*(1,69) = 15.919, *p* < 0.001, η^2^_p_ = 0.05), with an increment in the number of correct responses from the beginning to the end of the videogame administration in both HC (low: 78.7 ± 1.5; low_bis_: 79.5 ± 0.8) and pwMS (low: 77.5 ± 3.0; low_bis_: 78.9 ± 1.5). Notably, we found neither a main effect of *Group* (*F*(1,69) = 3.256, *p* = 0.08, η^2^_p_ = 0.05) nor of the interaction Time*Group (*F*(1,69) = 1.738, *p* = 0.19, η^2^_p_ = 0.006).

### 3.4. Correlations with the SDMT

Table 1 summarizes the pwMS’s performance on the SDMT.

None of the pwMS included in the present study were found impaired (i.e., z score < −1.64) on the SDMT administered at the time of videogame testing.

The correlation analyses between the SDMT and the videogame mean RTs showed a significant negative correlation between the total score on the SDMT and the mean RTs of level 1 (low; rho = −0.413; *p* = 0.004; CI [−0.62; −0.15]), level 2 (medium; rho = −0.395; *p* = 0.005; CI [−0.61; −0.13]), and level 3 (high; rho = −0.380; *p* = 0.008; CI [−0.60; −0.11]).

Similarly, significant positive correlations were found among the SDMT and accuracy of level 1 (low; rho = 0.410; *p* = 0.004; CI [0.14; 0.62]), level 2 (medium; rho =0.558; *p* < 0.001; CI [0.33; 0.73]), and level 3 (high; rho = 0.570; *p* < 0.001; CI [0.34; 0.74]).

## 4. Discussion

The primary aim of the present study was to assess the usefulness of a tablet-based videogame in assessing IPS in different conditions of increasing visual-attentional load in pwMS. The results highlighted that the videogame was able to detect a more pronounced IPS slowness as a function of cognitive load in pwMS as compared with HC. Indeed, even though pwMS were classified as having no CI and no IPS impairment on paper-and-pencil tests, they showed significant reduced performance on the videogame levels as compared with HC.

In both groups mean RTs significantly increased from the first to the third level, and the number of correct responses was significantly reduced in level 3 in comparison with the previous two levels. One might argue that the fixed-order presentation of the videogame levels, with the most difficult administered as last, might have led to a bias due to a confound effect of a “physiological” short-term depletion of cognitive resources over time. The results on the comparison between the low and the low_bis_ levels, however, showed that both pwMS and HC performance did not decrease from the beginning to the end of the videogame administration, as a progressive depletion of cognitive resources over time might have implied. Thus, the decrement in performance observed from the first to the third level can be attributed to the increasing visual-attentional load.

Crucially, pwMS seemed to be more susceptible to the increased visual-attentional load than the HC. PwMS were found to be, on average, slower than HC on the videogame levels; moreover, the decrement in accuracy through the levels was significantly more pronounced in pwMS than in HC. Looking separately at the single levels, we observed that the difference on mean RTs between the two groups was significant in the first two levels (i.e., low and medium) but not in the third (high) one. A specular result was found with respect to accuracy: a significant difference in the number of correct responses between pwMS and HC was found only in the third (high) level, with pwMS being significantly less accurate than HC. We argue that the results on mean RTs and accuracy should be interpreted as a whole. Specifically, each trial of each videogame level was characterized by a 1000 ms time limit that forced the participant to operate a choice: (1) to be accurate with the risk of not being able to answer on time, or (2) to be fast with the risk of making more errors. That considered, the cost that pwMS had to pay to be as accurate as HC on the first and the second levels was reflected in significantly slower mean RTs; vice versa to be approximately as fast as HC on the third level, pwMS had to significantly lower their accuracy. These results might be interpreted in the light of the speed–accuracy tradeoff (SAT) relationship [33]: when cognitive tasks stress speed, the responses are faster and, thus, less accurate; by contrast, when tasks stress accuracy, the responses are slower and more accurate. In this respect, several authors brought evidence in support to the notion that, usually, pwMS are able to achieve performance accuracy rates comparable to those of HC, but only if they are given more time to process the information (e.g., [34,35]). Notably, the SAT relationship is far from being absolute or linear and can be affected by different load conditions. With specific reference to MS, it has been shown that in low working memory load conditions pwMS are as accurate as HC, but slower; by contrast, in high working memory load conditions, also accuracy can be affected [36], evidence in line with the results of the present study.

Our results can also be interpreted in the light of the so-called “disconnection syndrome” [37,38] according to which demyelinated axons transmit signals at a slower pace resulting, on a behavioral level, in IPS deficits. In high-load conditions, the cognitive requests are higher, thus requiring the involvement of more widespread networks. In this context, the slowed myelin conduction, in association with neurodegeneration phenomena such as synaptic and dendritic loss, may contribute in unveiling an underlying reduced IPS [21]. Our results seem to be in line with this interpretation: pwMS were found to be more susceptible than HC to the cognitive load, as evidenced by the more pronounced decrement in accuracy as a function of the visual-attentional load associated with a concurrent slowness in mean RTs during the videogame levels.

The secondary aim of the present study was to evaluate the concurrent validity of the videogame in assessing IPS in pwMS. In this respect, a significant correlation was found among each videogame level and the SDMT, thus offering evidence in support of the videogame levels as specific measures of IPS in pwMS. The SDMT is a valid and reliable screening measure that has been recommended for use in both screening and monitoring perspectives in pwMS [39]. Similarly, our videogame might be potentially used as a brief screening tool to detect initial warning signals to be further investigated with a more extensive clinical evaluation.

The results of the present study fit well with the growing literature which is emphasizing the predominant role that computerized testing might assume in clinical practice [40]. Within this panorama, our videogame distinguishes itself by the fact that patients are tested while basically just *playing*. The framework of our videogame brings the same advantages of other computerized paradigms with respect to the high degree of precision and accuracy. However, in addition to that, the videogame format offers an inherent novelty and engaging playfulness not comparable to the setting of laboratory-based computer tasks. Videogames are easygoing and friendly tools which might allow for higher compliance in comparison with traditional tests, especially in the youngest patients. 

Despite the encouraging results, the present study is not free from limitations. First, the sample size is quite small and includes only patients with relapsing remitting course identified as cognitive-preserved on the paper-and-pencil tests. Future studies should include a bigger sample, representative of different forms of MS as well as of different degree of CI, to assess if the videogame is able to discriminate different MS phenotypes. Second, it is worth noting that a limitation of the videogame, for the way it is conceived so far, is that it requires the absence of visual and upper-limbs motor impairment, thus limiting its applicability to the general MS population. Third, administering the recommended gold standard tests also to a group of HC might help to evaluate further the sensitivity and the concurrent validity of the videogame. Fourth, the development of new scenarios is needed for the assessment of a broader range of cognitive components that might be early affected in the disease course. Lastly, further research over the videogame’s psychometric characteristics, validity, reliability, and usability needs to be conducted before releasing it into clinical practice. Specifically, important issues regarding its susceptibility to practice effects should be taken into account in the perspective of repeated administration to monitor patients’ cognitive functioning over time.

## 5. Conclusions

With the present study we evidenced that manipulating the visual-attentional load may account for a promising approach for the early detection of IPS inefficiency, even in apparently non severe and cognitive stable pwMS. Our videogame might, indeed, account for a useable screening tool for precisely estimating IPS functioning in pwMS. Video gaming is a popular form of leisure activity and its effect on cognition, as well as its usability in cognitive assessment, monitoring, and rehabilitation, has come into increasing interest in the past few years [14,41]. Future studies should emphasize the high potential that might derive from the adoption of computerized assessment tools in clinical practice in pwMS, especially with reference to monitoring disease progression over time.

## Figures and Tables

**Table 1:** PwMS performance on the Symbol Digit Modalities Test (SDMT).

	Raw Scores Mean ± SD (Range)	*Z-Scores*					
		Less than −2.0 SD	Less than −1.64 SD (Cut-Off)	Less than −1.5 SD	Less than −1.0 SD	Between −1.0 and 0.0 SD	More than 0 SD
SDMT	59.3 ± 13.1 (40–110)	0	0	0	1	14	36
